# Case Report: Vitreous Amyloidosis Caused by a *TTR* Lys55Asn Mutation With Intraoperative Suprachoroidal Hemorrhage

**DOI:** 10.3389/fmed.2021.797223

**Published:** 2022-01-24

**Authors:** Qing Xu, Xinting Wang, Zhengpei Zhang, Jie Li, Haiyang Liu, Sujuan Ji, Lei Qiao, Chaoju Gong, Ruifang Feng, Suyan Li

**Affiliations:** Department of Ophthalmology, The Affiliated Xuzhou Municipal Hospital of Xuzhou Medical University, Xuzhou First People's Hospital, Xuzhou Eye Disease Prevention and Treatment Institute, Xuzhou, China

**Keywords:** vitreous amyloidosis, *TTR*, Lys55Asn mutation, vitreous opacity, suprachoroidal hemorrhage, vitrectomy, case report

## Abstract

We report a vitreous amyloidosis patient with white vitreous opacities (footplates) adhering to the posterior lens capsule. A positive Congo-red stain and transthyretin (*TTR*) Lys55Asn mutation confirmed the diagnosis of vitreous amyloidosis. Optical coherence tomography (OCT) revealed fern-like material adhering to the the posterior pole retinal surface in both eyes. Visual acuity significantly improved after the first vitrectomy, but vitreous opacities recurred 4 years later. The patient appeared to have aggravated sensorimotor neuropathy and severe autonomic dysfunction at the same time. He developed intraoperative suprachoroidal hemorrhage during the second vitrectomy.

## Introduction

Familial amyloidosis polyneuropathy (FAP) is an autosomal dominant inherited disorder caused by a mutation of the transthyretin (*TTR*) gene and it manifests with either systemic symptoms or local vitreous amyloidosis (1, 2). The *TTR* protein is synthesized not only in the liver but also in the retinal pigment epithelium and choroid plexus (3). Amyloid has an affinity for basement membranes and it can also deposit on the lens, iris, trabecular meshwork, or within the walls of retinal and choroidal vessels (4). The well-described clinical signs of vitreous amyloidosis include glass wool vitreous, perivascular deposits, or opacities attached to the posterior lens capsule, which are referred to as *pseudopodia lentis* (5). Diagnosis is based on clinical suspicion, Congo-red staining, and *TTR* gene sequencing (6). Vitrectomy may significantly improve visual acuity, but the amyloid deposits may recur because of residual vitreous opacities (3, 7). Here, we describe a recurrent vitreous amyloidosis patient with a *TTR* Lys55Asn mutation who presented with intraoperative suprachoroidal hemorrhage during the second vitrectomy.

## Case Description

**Figure 1:**
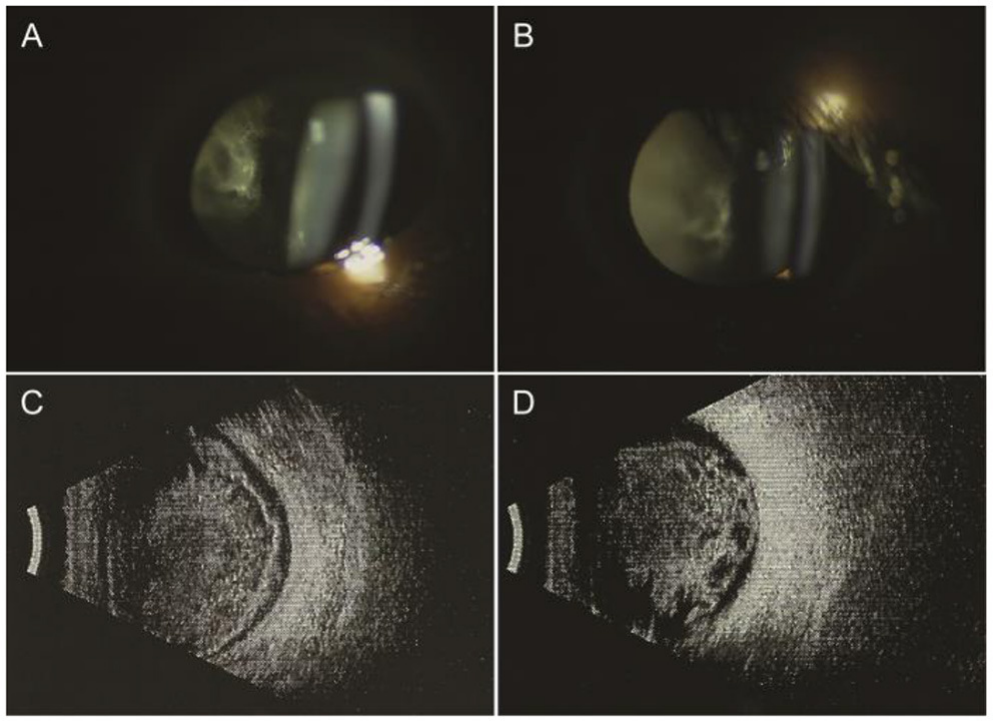
Ophthalmic examination before the first operation. Anterior segment examination showed white vitreous opacities (footplates) adhering to the posterior lens capsule. B-scan ultrasonography revealed that the glass-wool vitreous opacities were almost completely detached except for the local adhesion to the retina and optic disc. **(A,C)** Right eye; **(B,D)** left eye.

**Figure 2:**
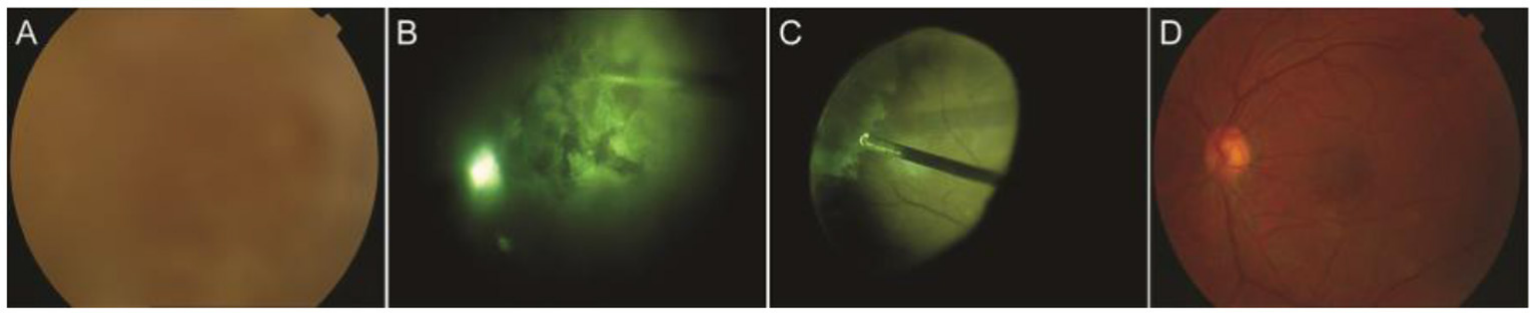
Images of the funds and the first operation. **(A)** Color fundus image was very blurred before vitrectomy in the left eye; **(B)** Fibrillary and stiff vitreous opaque in the right eye; **(C)** As much of peripheral vitreous was removed as possible; **(D)** Normal fundus in the left eye 1 week after vitrectomy.

A 50-year-old male presented with a 5-year history of painless decreased vision and a 1-year history of rapid visual deterioration. He suffered from poliomyelitis since childhood, with difficulty in walking on one lower limb and pericardial effusion. He was previously diagnosed with bilateral uveitis and did not respond to corticosteroid treatment. On examination, visual acuity was hand motion in both eyes. Intraocular pressure was normal. Slit-lamp biomicroscopy showed no alterations in the anterior segment. The light pupillary reflex was normal. The anterior segment examination showed white vitreous opacities (footplates) adhering to the posterior lens capsule (Figures 1A,B). The B-scan ultrasonography revealed that the glass-wool vitreous opacities were almost completely detached except for the local adhesion to the retina and optic disc (Figures 1C,D). The color fundus image was very blurred, showing no details of the fundus (Figure 2A).

The patient received a 23-G pars plana vitrectomy in the right eye first, and then in the left eye 7 months later. The right eye had a severe vitreous opacity with no red reflex during the surgery. The opaque vitreous was fibrillary and stiff (Figure 2B). The peripheral vitreous was removed as much as possible during the vitrectomy (Figure 2C). The characteristics of the vitreous opacity in the left eye were the same as for the right eye. Visual acuity was 1.0 in both eyes 4 months after surgery. The lenses were transparent and the vitreous was clear. Color fundus photography revealed a normal retinal vasculature (Figure 2D).

**Figure 3:**
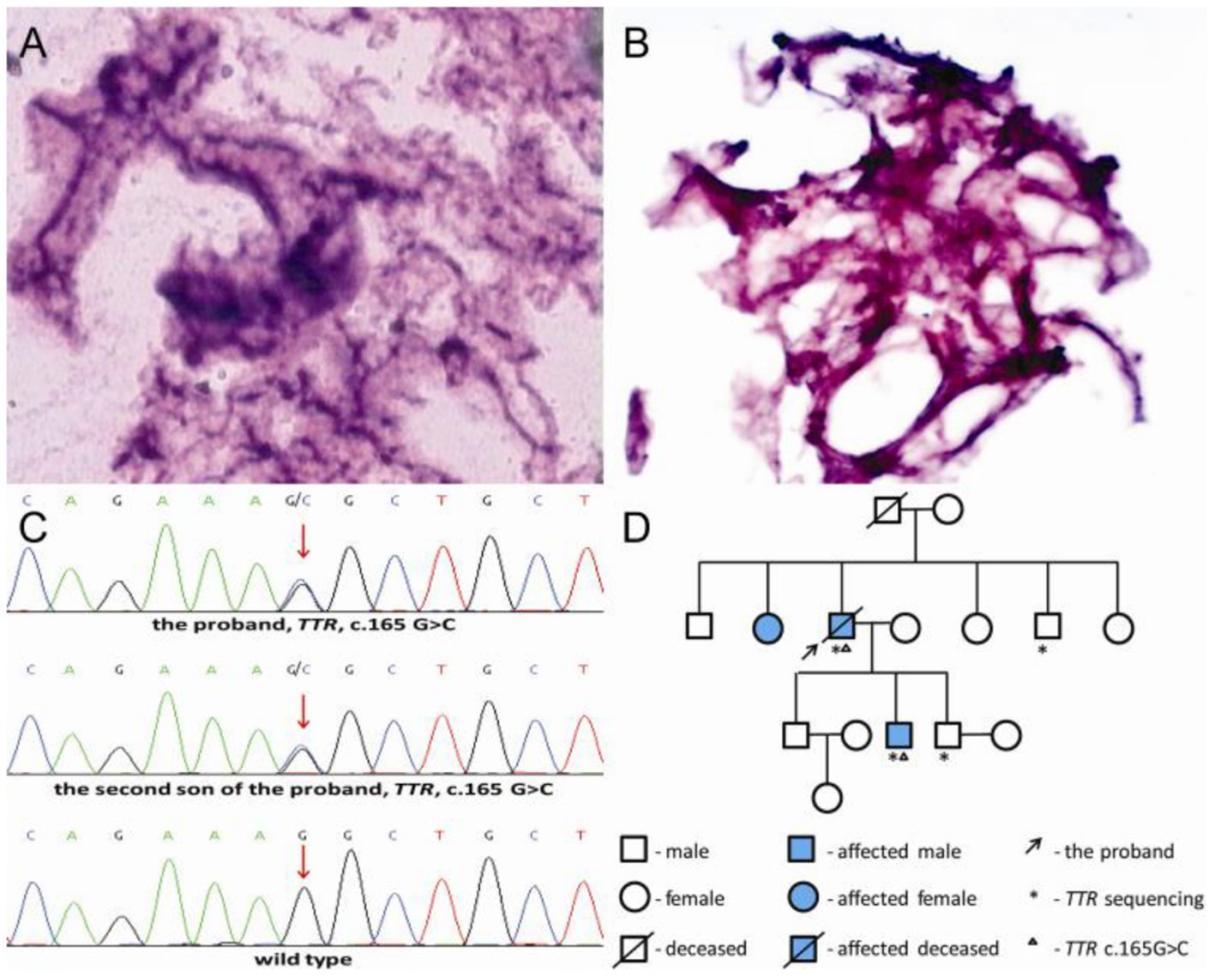
Histopathological examination and transthyretin (*TTR*) gene sequencing. **(A)** Hematoxylin-eosin (H&E) staining revealed degeneration vitreous; **(B)** Congo-red staining positive revealed amyloid vitreous; **(C)** The patient was heterozygous for a *TTR* c.165 G>C mutation and his second son carried the same mutation; **(D)** Pedigree of the family.

Histopathological examination revealed acellular eosinophilic materials with hematoxylin-eosin (H&E) stain (Figure 3A) and a very strong positive red stain of the vitreous amyloid with Congo-red stain (Figure 3B). Genetic tests showed that the patient was heterozygous for a *TTR* gene single nucleotide substitution c.165 G>C in exon 2, resulting in the replacement of lysine with asparagine at position 55 of the mature protein, that is, p.Lys55Asn (Figure 3C). The proband's two sons and brother received pedigree testing, which revealed that the second son carried the same mutation and the others were normal (Figures 3C,D). Ophthalmologic examination showed that both eyes of his second son were normal. However, he developed autonomic neuropathy with delayed gastric emptying and gastrointestinal dysfunction as the first symptom of FAP at 30 years old. B-scan ultrasonography showed snowball-like vitreous opacities in both eyes of the proband's sister who refused to accept pedigree testing.

**Figure 4:**
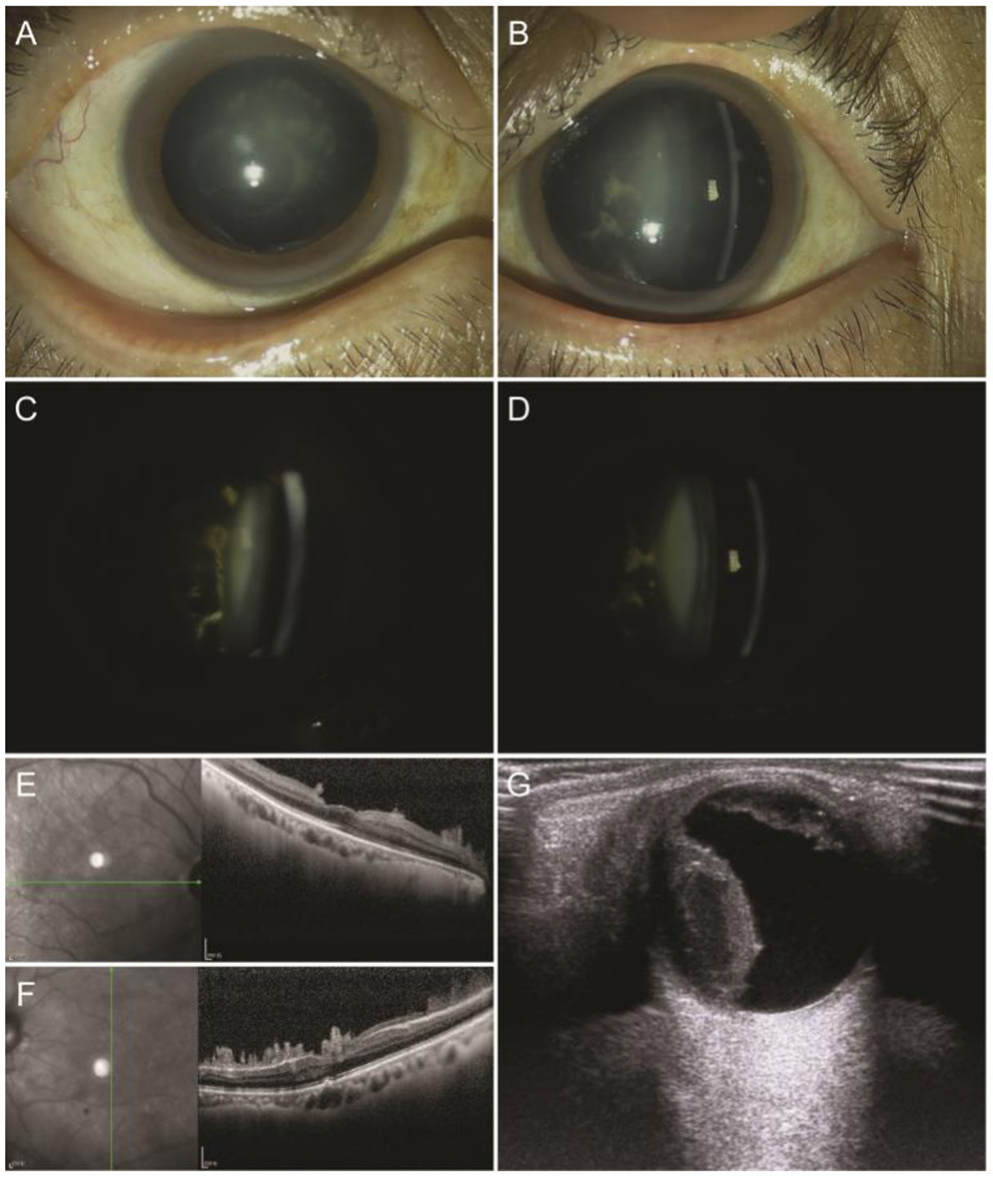
Images of the recurrent vitreous amyloidosis. Anterior segment images showed opaque nuclear with white heterogeneous opacity adhered to the posterior lens capsule. Optical coherence tomography (OCT) showed fern-like material adhering to the posterior pole retina surface. **(A,C,E)** Right eye; **(B,D,F)** left eye. **(G)** Color-Doppler ultrasound showed a dome shape temporal elevation of the retina with increased echo density in the right eye.

Four years later, the patient complained of a vision decrease in both eyes associated with the inability to walk on his lower limbs for half a year. A physical examination revealed anemia, muscle atrophy and paralysis of both lower limbs, inability to walk, emaciation, deafness, diarrhea, urinary incontinence, and urinary retention. Upon ophthalmologic examination, visual acuity was counting fingers in the right eye and 0.1 in the left eye. The lens nucleus was opaque in both eyes (nuclear sclerosis grade of 2), with white heterogeneous opacities adhered to the posterior lens capsule (Figures 4A–D). Color fundus photography revealed blurriness of the right fundus and only faintly visualized fundus color in the left eye. Optical coherence tomography (OCT) revealed fern-like material adhering to the posterior pole retinal surface in both eyes (Figures 4E,F).

He was hospitalized in the urological ward for urinary incontinence, during which an upper gastrointestinal tract hemorrhage occurred and was treated successfully. An anticoagulant test showed no abnormal changes. Vitrectomy combined with phacoemulsification on his right eye was performed after his general condition became stable. A brownish dome shape elevation of the temporal retina was noticed intra-operatively, and the operation was terminated due to concern of a possible suprachoroidal hemorrhage.

No obvious discomfort was observed after surgery. Intraocular pressure was normal. Slit-lamp biomicroscopy showed some blood accumulation in the anterior chamber and the white retrolental opacities were no longer visible. There was moderate hemorrhagic turbidity of the vitreous that precluded visualization of the fundus. A dome shape temporal elevation of the retina with increased echo density was noted with a color Doppler ultrasound (Figure 4G). The patient appeared to have transient hypotension syncope after position changes. Color echocardiography showed thickening of the general left ventricle, limited movement, decreased systolic and diastolic function of the left ventricle, and pericardial effusion. An abdominal color ultrasound showed dilated hepatic veins, hydronephrosis of both kidneys, and dilated bilateral ureters. Cardiology, gastroenterology, neurology, and the pharmacy were consulted and the patient was confirmed to have severe malnutrition, ventricular hypertrophy, and very poor cardiac function. Conservative medical management and no additional surgery were recommended. The patient died of sudden heart failure 3 months later.

## Discussion

We report a rare case of Congo-red positive staining and a *TTR* Lys55Asn mutation related to vitreous amyloidosis. The patient had a typical retrolental change with adherent footplate-like opacities, a glass-wool appearance of the vitreous opacity, and a typical OCT change of fern-like material adherent to the posterior retinal surface (8, 9). He had no history of glaucoma, conjunctival changes, corneal opacity, or pupillary abnormality. The initial vitrectomy resulted in the excellent recovery of the visual acuity. The histopathology of the vitreous specimen with Congo-red stain and the genetic test were essential in the confirmation of the diagnosis of vitreous amyloidosis.

Kleefeld et al. reported that subarachnoid hemorrhage can be caused by cerebral amyloid angiopathy. Amyloid deposits in the cortical and subcortical blood vessels can cause vascular endothelial dysfunction, increased brittleness of vessels, destruction of small vessels, and ultimately, bleeding (10). Although suprachoroidal hemorrhage tends to happen more frequently during intraocular surgeries following the previous vitrectomy due to the lack of vitreous support and easier fluctuation of intraocular pressure, we speculate that amyloid induced vasculopathy in the choroid could also be a triggering factor to induce the suprachoroidal hemorrhage with the same pathophysiology of brain vascular changes. Amyloid retinal and choroidal angiopathy tends to appear late in the course of the disease, and choroidal amyloid depositions were frequently observed in the choroidal arterial vasculature (11, 12). Poignet et al. reported three patients with vitreous amyloidosis who all had choroidal angiopathy in late indocyanine green angiography (ICG) (13). It is possible that abnormal choroidal vessels are due to direct infiltration with amyloids or secondary to underlying cardiac problems (7). Hence, it may be valuable to conduct ICG to assess the choroidal vascular changes as a risk factor before a surgeon performs a second vitrectomy for a visually significant vitreous amyloidosis patient.

## Data Availability Statement

The original contributions presented in the study are included in the article/supplementary materials, further inquiries can be directed to the corresponding author/s.

## Ethics Statement

Written informed consent was obtained from the individual(s) for the publication of any potentially identifiable images or data included in this article.

## Author Contributions

QX: report explanation, patient workup, figure preparation, and manuscript preparation. XW, ZZ, JL, HL, and SJ: patient workup and manuscript revision. LQ, CG, and RF: manuscript revision. SL: report explanation, patient workup, figure preparation, and manuscript revision. All authors contributed to the article and approved the submitted version.

## Funding

This study was supported by the Xuzhou Medical Outstanding Personnel Plan (XWJC 001).

## Conflict of Interest

The authors declare that the research was conducted in the absence of any commercial or financial relationships that could be construed as a potential conflict of interest.

## Publisher's Note

All claims expressed in this article are solely those of the authors and do not necessarily represent those of their affiliated organizations, or those of the publisher, the editors and the reviewers. Any product that may be evaluated in this article, or claim that may be made by its manufacturer, is not guaranteed or endorsed by the publisher.
